# Estimation of Ion Competition via Correlated Responsivity Offset in Linear Ion Trap Mass Spectrometry Analysis: Theory and Practical Use in the Analysis of Cyanobacterial Hepatotoxin Microcystin-LR in Extracts of Food Additives

**DOI:** 10.1155/2013/414631

**Published:** 2013-03-26

**Authors:** Jan Urban, Pavel Hrouzek, Dalibor Štys, Harald Martens

**Affiliations:** ^1^Laboratory of Applied System Biology, Institute of Complex Systems (Former Institute of Physical Biology), South Bohemian Research Center of Aquaculture and Biodiversity of Hydrocenoses, Faculty of Fisheries and Protection of Waters, University of South Bohemia in České Budějovice, Zámek 136, 37333 Nové Hrady, Czech Republic; ^2^Nofima-Norwegian Institute of Food, Fisheries and Aquaculture Research, Osloveien 1, 1430 Ås, Norway; ^3^Department of Phototrophic Microorganisms, Institute of Microbiology, Academy of Science of the Czech Republic, Opatovický mlýn, 373 81 Třeboň, Czech Republic

## Abstract

Responsivity is a conversion qualification of a measurement device given by the functional dependence between the input and output quantities. A concentration-response-dependent calibration curve represents the most simple experiment for the measurement of responsivity in mass spectrometry. The cyanobacterial hepatotoxin microcystin-LR content in complex biological matrices of food additives was chosen as a model example of a typical problem. The calibration curves for pure microcystin and its mixtures with extracts of green alga and fish meat were reconstructed from the series of measurement. A novel approach for the quantitative estimation of ion competition in ESI is proposed in this paper. We define the correlated responsivity offset in the intensity values using the approximation of minimal correlation given by the matrix to the target mass values of the analyte. The estimation of the matrix influence enables the approximation of the position of *a priori* unknown responsivity and was easily evaluated using a simple algorithm. The method itself is directly derived from the basic attributes of the theory of measurements. There is sufficient agreement between the theoretical and experimental values. However, some theoretical issues are discussed to avoid misinterpretations and excessive expectations.

## 1. Introduction

 Mass spectrometry connected with high-performance liquid chromatography (HPLC-MS) is a widely used analytical tool for the analysis of complex biological samples and the detection of different kinds of organic compounds. Recently, the potential of HPLC-MS for metabolomic studies has been highlighted due to its capability of routinely handling large sequences of samples. This instrument provides excellent reproducibility and usefulness for qualitative analysis. However, some questions have been raised about the quantitative abilities of HPLC-MS analysis. Several studies have discussed the fact that ion competition among different analytes exists when they are simultaneously ionized [1–5]. The molecules eluting from the HPLC column are ionized at the MS device. There is not equal probability for all molecules to be ionized. The ionization itself depends on many physicochemical factors. Some molecules are just much easier to ionize than others. This process is called ion or charge competition. However, the extent of the uncertainty, which highlights the potential impact of ion competition on the analysis of complex biological samples, has not yet been given sufficient attention. Traditionally, triple quadrupoles have been prefered for quantitative analyses. However, recently it has been shown that linear ion traps can be rather more appropriate in certain analytes such as mixtures of specific peptides in biological specimens [6]. 

 The effectiveness of an analysis depends on two key features of the measurement: (i) experimental performance by the operator and (ii) performance of the instrument. The first feature comprises the precision of the operator during sample preparation as well as during the measurement itself. The second feature may be characterized by the proper mathematical description of the measurement device attributes according to the theory of measurement (general descriptions of basic attributes of every measurement device), which is done to encapsulate the analysis into the appropriate mathematical space. The layout of the possible domain values ensures that the interpretation of the measured datasets also fulfills the mathematical presumptions of the measurement process. Unfortunately, this point of view is not often practically supported.

 The basic attribute of the measurement is responsivity. As was already pinpointed in the literature [7, 8], there are several mutually interchanged definitions of responsivity, sensitivity, and limit of detection. Generally, the responsivity is a conversion qualification of the measurement device given by the functional dependence (transfer function) between the input and output quantities. Then the sensitivity is the minimum magnitude of input signal required to produce a specified output signal, and it is related to the standard deviation of the measured value [8]. In other words, sensitivity is a number, while responsivity is a function. In HPLC-MS, there are three individual quantities for which responsivity should be examined: (i) retention time (rt), (ii) mass to charge ratio (*m/z*), and (iii) intensity, which represents the amount of ionized molecules of individual *m/z*'s at an exact discrete time point or rt.

 The retention time is determined by the separation process on the chromatographic column. The responsivity of the rt quantity is therefore based on the sampling frequency, gradient time, peak capacity, column temperature, and flow rate. The mass to charge ratio depends on the MS detector accuracy and precision and, therefore, on the resolution (or distinguishability). The intensity values for every individual measurement run are generated for all possible pairings of rt *m/z*. Thus, mathematically, the intensity is a set of natural numbers including zero. The maximal value of the intensity set is delimited by the saturation level of the MS detector and is called the mass limit. In this paper, we focus on the responsivity and deduce an attribute limit of detection for the intensity quantity. The most simple experiments for responsivity testing are calibration curves (the second most often are so-called contrast curves, where actual values are not important; however, the relative changes inside groups of values in repetitions are used for nonlinearity and heteroscedasticity tests). 

 One of the most used representations of measurement capabilities is the limit of detection, which is related to the responsivity, as will be shown later. The limit of detection is usually expressed as the lowest concentration or amount of the analyte that can be clearly detected with a stated degree of reliability from the background or blank sample. However, the evaluation of LOD in concentrations is just a recommendation; generally the formula is valid even for intensity units, the interpretation of LOD is then slightly different as will be shown, but for our purpose it remains consistent. The blank sample is a sample that does not contain analyte but has a matrix that is identical to that of the average analyzed sample. Therefore, the limit of detection (LOD) should vary in different matrices. To verify this, two different matrices of food additives, that is, filamentous green alga *Stigeoclonium* sp. and salmon meat hydrolyzate were tested. In order to test the abilities of HPLC-MS as a detection tool, we analyzed complex samples of food additives with known amounts of added microcystin-LR (MCYST-LR). In this study, we also tested if and to what degree other compounds affect the responsivity to MCYST-LR in the mass spectrometry measurement.

 MCYST-LR is heptapeptide with a molecular weight of 994.5 Da that is produced by different cyanobacterial taxa, for example, *Microcystis*, *Nostoc*, *Anabaena*, and so forth [9–13]. It was proven that MCYST-LR causes hepatosis via the inhibition of protein phosphatases in the liver cells of mammals including humans [14, 15]. Due to the ability of cyanobacteria to form heavy water blooms, the negative effects of MCYST-LR and other microcystins represent significant problems for drinking water supplies. Moreover, the stability of microcystin implies that it can accumulate in high concentrations in fish organs [16]. Another potential problem is the easy air transportation of the toxigenic cyanobacteria, which can result in the contamination of microalgae being harvested for use as food additives. The microcystin problem was addressed by the World Health Organization, which recommended a safe concentration of 1 *μ*g/L of microcystin in drinking water. Current methods for microcystin monitoring are mainly based on HPLC measurements with UV detection, or by specific protein phosphatase inhibition tests, that is, PPI [17] and ELISA tests [18, 19]. The HPLC-UV method suffers due to a lack of sensitivity towards low concentrations. Although the PPI test and ELISA are sufficiently sensitive, alternatively, LC in tandem with mass spectrometry (HPLC-MS) offers a robust method with, in principle, sufficient sensitivity. Thus, HPLC-MS was applied to the detection of MCYST in water and cyanobacterial biomass [20, 21] with sufficient sensitivity and reproducibility. Karlsson and others [21] tested two MS instruments for the quantification of MCYST-LR in mussels and flounder extracts. They concluded that MS provides a good toll with linear response and that it is more appropriate for the analysis of MCYST-LR content in tissue samples than the ELISA method [21]. The sensitivity and linear fitting of the calibration curves were better for the triple-quadrupole MS than the ion trap MS [21].

 The aim of the present study is to characterize and describe the responsivity and minimal ion suppression in mass spectrometer with linear ion trap. We focus on three connected subtopics: (i) the determination of the responsivity function for intensity values in HPLC-MS, (ii) the discussion of the LOD and its statistical interpretation, and (iii) an estimation of the ion competition in different biological matrices using the knowledge of responsivity.

## 2. Experimental

### 2.1. Materials

 The calibration curve for pure MCYST-LR was constructed from the analysis of 10 MCYST-LR concentration measurements. The pure MCYST-LR standard (Sigma no. 33893) was diluted in methanol to obtain the required concentrations. Two food additives were mixed with known concentrations of MCYST-LR: (i) filamentous green algae *Stigeoclonium* sp. (obtained from BP Medical, Brno, Czech Republic) and (ii) salmon meat hydrolyzate (Nofima Mat, As, Norway). The food additive samples were both extracted in 70% MeOH at an extraction ratio of 200 mg of biomass per 10 mL of 70% MeOH and centrifuged (4000 rpm, 15 min). The supernatant was then removed and the sample was concentrated on a C8 HLB Cartridge (Waters Oasis) into 1 mL of MeOH. The final extracts were analyzed both without and with the addition of a known amount of MCYST-LR.

### 2.2. Instrumentation

 The extract composition was analyzed using an HP 1100 Agilent liquid chromatogaphy with an HP 100 XD SL-Ion trap. The extract was separated on a reversed phase column (Zorbax XBD C8, 4.6 × 150 mm, 5 *μ*m) at 30°C and eluted by gradient MeOH/H_2_O + 0.1% HCOOH (30–100% MeOH for 30 min, 100% for 5 min) at a flow rate of 0.6 mL/min. The settings of the electrospray ionization were as follows: positive mode, ramp range from 1500 to 4500 V, nebulizer at 50 psi, dry gas at a flow rate of 10 L/min, and a dry temperature of 325°C. The ion trap was set to target mass 900 with a range of 100 to 1000 in profile mode. The obtained total ion chromatograms (TICs) were evaluated and protonated molecular ions were detected on the basis of signal intensity, presence of sodium and potassium adducts, and distribution of isotopologues. 

### 2.3. Methods

 Obtained data were processed using both manual (supervised) and automatic (unsupervised) tools to compare the two approaches. The analysis of the pure MCYST-LR calibration and mixed samples was carried out to evaluate the influence of food additives extracts on the quantitative responsivity of the mass spectrometer. Supervised parametrized analysis was carried out using the Bruker Daltonik software DataAnalysis 3.3 for data obtained using the LC/MSD trap, which is the standard tool used for the Agilent device. Raw TICs were preprocessed for peak integration in Data Analysis using supervised parameters. The parameters used consist of a Gaussian smoothing of width equal to 4 points in 2 cycles. The outputs of the supervised analysis were exported as Cmpd Mass Spec List Report—MS (P) Layout and contained information on the retention time, maximal intensity, and area after smoothing. The unsupervised nonparametric analysis was carried out using EMP (Expertomica Metabolite Profiling) [22] as a Matlab Runtime stand-alone application. This software automatically removes random noise and baseline contributions according to the probabilistic behavior and separate measurements of compounds. The results of the unsupervised analysis were outputted as PRT ASCII tables of segmented compounds and included the retention times, maximal intensities, areas, confidential factors, and basic statistical evaluations.

 The outputs of both software, that is, Bruker Daltonics DataAnalysis, and EMP were used for comparison of the selected protonated MCYST-LR molecular ion and doubly charged sodium adduct of the molecular ion peaks. The responsivity (dependency of the detector response on known concentrations) for pure and mixed MCYST-LR samples was fitted. The fitting process was carried out for maximal intensity and for the area values of the selected peaks using Matlab cftool. An evaluation of the measurement attributes (responsivity, LOD, CRO, correlation coefficients) was also performed in Matlab.

*(i) Responsivity Function*. The responsivity is defined as the ratio between the output signal, *y*, and the measured property, *c*. Ideal dependence is given by a linear function: *y* = *s*∗*c* + *o*, where *c* is the concentration, *y* is the detector response (maximal intensity or area), *s* is the slope of linear curve, and *o* is the offset in the measurements of the calibration curves. The slope of the linear curve is equal to the responsivity of the measurement and is also a parameter of a transfer function. It is constant only in linear cases. 

 Generally, the responsivity is defined as a derivative of the transfer function: *K* = lim⁡*dy*/*dc* and therefore depends on the measured value. The derivative is the local slope of the transfer function. Typical transfer functions are logarithmic or exponential, which applies even to the human perception of light intensity and sound. Therefore, the first practical consideration is which type of transfer function should be used to determine the calibration curves for HPLC-MS. 

 The most puzzling issue is the task of the fitting function type specification [23–25], that is, the search for data processing that constructs mathematical mapping that minimizes the displacement of the data points. Our classes of possible functions comprise polynomial functions of first degree (linear) and exponential functions.

*(ii) Limit of Detection*. Another question is how a matrix used influences the responsivity. One of the characteristic parameters of matrix influence is the limit of detection (LOD). LOD is defined by the IUPAC GoldBook [26] as the mean blank value plus *k* times the standard deviations, where *k* is a numerical factor chosen according to the confidence level desired. In the past, IUPAC has recommended a value of *k* = 3 [7]. The rationale is that the standard deviation for the blank sample is roughly equivalent to the standard deviation for small concentration of analyte [27–29]. However this approach was criticized by Needleman and Romberg [29], because the LOD represents the average noise and defines only the ability to measure nothing. We agree with the latter viewpoint. However, we cannot simply refuse the statistical approach as a whole.

 The limit of detection is often incorrectly [27] called the sensitivity. However, sensitivity is the minimum magnitude of input signal required to produce a specified output signal. It is usually assumed to be equal to the root mean square deviation of the sensor noise. When an analyte is mixed with a matrix, the standard deviation of the matrix blank can be used as an estimation of the sensitivity. The rationale for this assumption is as follows: the mean value of the matrix blank contributes to the responsivity offset of the relevant analyte signal. It is necessary for the analyte signal to be higher than that of the blank by at least as much as the standard deviation of the blank matrix to be able to recognize changes in the analyte signal. Much has already been written about the subject of limits of detection [7]. The LOD computed via the mean blank value and *k* times the standard deviation is defined with respect to univariate calibration. Multivariate methods were described by Garner and Robertson, van der Voet, Olivieri et al., and Boqué et al. [30–33]. An approach using multivariate detection limits (MDLs) was developed by Boqué et al. 1999 [33]. The real sensitivity value is therefore also multivariate dependent, which must be taken into account.

 The detection decision at the LOD leads to a risk of false detects. The LOD is constructed as the level of false nondetects with some probability. This definition offers the possibility to detect an analyte below the LOD because the proper values of risk and probability are sample dependent. Hypothesis testing involves the distribution of results under the null hypothesis only. The probability of false nondetect increases with decreasing analyte concentration. However, the risk of false positive remains small as long as the result exceeds some critical level. The IUPAC definition of LOD is based on the homoscedastic assumption that the uncertainty does not depend on the actual analyte level. This assumption is usually violated [33].

 In this paper, we present a definition of the correlated responsivity offset (CRO) of detection, which is derived from the common interpretation of the LOD. The connection of HPLC with MS is advantageous in comparison to other chromatographic methods, as MS adds an extra dimension to the measured dataset. Therefore, the technical operating parameters of mean values and standard deviations should be computed independently for each measured *m/z*. Thus, we may obtain different LOD values for every single mass value in the blank measurement. Those LODs were computed independently. We also know that the biological matrix used somehow influences the variety of intensity values and, therefore, the calculated LODs. The reason for independent computation for each mass value is obvious. We want to investigate the properties of many variables. Global computations are always dangerous, because we are losing some details.

 In statistics, covariance provides a measure of the correlation between the changes of two variables. The covariance of two random variables is evaluated as the difference of the mean value of the multiplicity of the variables minus the multiplicity of the mean values of the variables. Variance of one random value is a special case of covariance and is used when the two variables are identical. The standard deviation is the square root of the variance. 

 The evaluation of many variables produces a covariant matrix in which the elements represent the covariance between two given variables. The most familiar measure of dependence between two variables is the correlation coefficient. The correlation coefficient is computed from standardized random variables, that is, the covariance of multiple variables' standard deviations. The square of the correlation coefficient times 100 is called the strength of relation, in percentage. 

 The total covariance of many variables is simply approximated as the maximal covariance of a given variable with all other variables. This approximated total covariance is always a bit lower than the true total covariance. Therefore, the maximal covariance of two variables is the minimal covariance of all variables. Correlated standard deviation should be also approximated as the square root of the approximated total covariance value.

 We therefore define the correlated responsivity offset (CRO) as the mean blank intensity value of a target *m/z* plus the correlated standard deviation. Our criticism of LOD is focused mainly on its interpretation. For both experimental and theoretical reasons, it is obvious that intensity values are often measured below the theoretical LOD. This is especially evident in calibration curves where these small values continue in the trend of the curve (see Section 3) and should undoubtedly be considered relevant. The reason, but not full justification, for this misinterpretation is that total ion chromatograms (TIC) feature small peaks that are hidden in the noise and mainly contribute to the baseline. This is a result of a strong magnification of the noise level by summing on the mass axis in TIC. However, in single-ion chromatograms (SIC) of a target mass, small peaks are occasionally revealed below the theoretical LOD. This confusion in the interpretation does not mean that there is no limit to the values that can be detected, it simply means that the description of the LOD value (defined via the mean value and standard deviation) as the smallest feasible detected value is unfortunate. A more intuitive interpretation is that the LOD value represents the responsivity offset (also correlated) in the intensity values, which are correlated, in relation to some “ideal” intensity values (noncorrelated). We will use this “offset” interpretation during the estimation of ion competition. That is the main reason, why we used the LOD expressed in intensity units instead of concentrations.

*(iii) Estimation of Ion Competition*. The biological matrices used affect the responsivity of the target mass. In other words, the detector response (output) for a given concentration (input) differs in different matrices. Let us suppose a linear responsivity function for an illustration of the estimation of the relationship between two responsivities.

(1) We measure two blanks of the matrices, *B*1 and *B*2, without the analyte.
(2) From each blank, we compute several statistical attributes of the target mass *M*, including the mean values, maximal covariances, correlated standard deviations, and correlation coefficients, *R*1 and *R*2 (as described in previous section).
(3) The mean values (2) and correlated standard deviations (2) are used for the evaluation of correlated responsivity offsets, CRO1 and CRO2 (defined in previous section).
 The measured mass *M* in the blanks is the same value of m/z as the value of the target (analyte) mass *M*. The analyte of mass *M* is not presented in the blanks. We are not computing the exact influence of the analyte in the blanks. Instead, we are computing the approximation of the influence of the matrix on noise with a similar m/z value. CROs represent the correlated responsivity offsets in the matrices for mass *M*. Correlation coefficients *R*1 and *R*2 represent the minimal correlations in the matrices for mass *M*.
(4) We also measure the calibration curve of mass *M* in matrix *B*1 via the dependency of the intensity *Y*1 (of target mass *M*) on the concentration *c*.
(5) The hypothetical linear responsivity function is *Y*1 = *s*1∗*c* + *o*1, where *s*1 is the responsivity slope in matrix *B*1 and *o*1 is the responsivity offset in matrix *B*1. The values of parameters *s*1 and *o*1 are fitted from the measurements of the *Y*1 dependency on *c*.
 We want to know how the calibration curve of target mass *M* in matrix *B*2, that is, the dependency of the intensity *Y*2 on concentration *c*, will look.
(6) The correlation coefficient is described as a slope between correlated variables. We can assume some “ideal curve” *X* of some independent variable that correlates with the target mass *M*. This ideal curve for matrix *B*1 is given as *X* = *R*1∗*Y*1.
(7) The same assumption as in previous step (6) is made for matrix *B*2: *X* = *R*2∗*Y*2.
(8) From the hypothetical responsivity (5) and the ideal curve (6), both in matrix *B*1, we estimate the ideal curve as *X* = *R*1∗(*s*1∗*c* + *o*1).
(9) Accordingly, we put the estimated ideal curve (8) into the equation for an ideal curve (7) in matrix *B*2 to obtain *Y*2 = (*s*1∗*c* + *o*1)∗(*R*1/*R*2).
(10) There remains the question of the proper offset in matrix *B*2. Our CRO values (3) are the estimations of the offsets in related matrices *B*1 and *B*2. The offsets are also correlated by the same correlation coefficients (3), *R*1 and *R*2.
(11) The final equation for the estimation of the calibration curve in matrix *B*2 is as follows:
  (1) $$\begin{array}{c} \;Y2=\left(s1*c+o1+\text{CRO}2-\text{CRO}1\right)*\left(\frac{R1}{R2}\right). \end{array}$$

This equation evaluates the minimal competition for target mass *M* in matrix *B*2. Of course, the competition should be slightly different. Essentially, the correlation coefficients will be higher as *R*1 and *R*2 are the minimal approximations of the total correlations and not the real total correlation coefficients. Therefore, the real *R*1/*R*2 ratio will be either lower or higher depending on the exact values of both total correlation coefficients.

 For the simple estimation of calibration curve *Y*2, only a few parameters are necessary: the correlation coefficients *R*1 and *R*2; the correlated responsivity offsets CRO1 and CRO2 from the blank measurements; and the fitting parameters *s*1 and *o*1 of the responsivity function in matrix *B*1. The estimated linearization of the calibration curve in matrix *B*2 has a responsivity slope given by responsivity slope *s*1 in matrix *B*1 modified by the ratio of the correlation coefficients (*R*1/*R*2). The estimated responsivity offset in matrix *B*2 is given by the responsivity offset *o*1 in matrix *B*1 and the correlated responsivity offsets CROs and is again modified by the ratio of the correlation coefficients (*R*1/*R*2). Therefore, the estimated linearization of the calibration curve in matrix *B*2 is

(2) $$\begin{array}{c} Y2=s2*c+o2, \end{array}$$

where *s*2 is the responsivity slope in matrix *B*2:

(3) $$\begin{array}{c} s2=s1*\left(\frac{R1}{R2}\right), \end{array}$$

and *o*2 is the responsivity offset in matrix *B*2:

(4) $$\begin{array}{c} o2=\left(o1+\text{CRO}2-\text{CRO}1\right)*\left(\frac{R1}{R2}\right). \end{array}$$

All values, as well as the estimation of calibration curve *Y*2, have to be computed independently for all target *m/z* values. The errors between the estimated and real calibration curves are explained in the Results and Discussion section.

## 3. Results and Discussion

**Figure 1:**
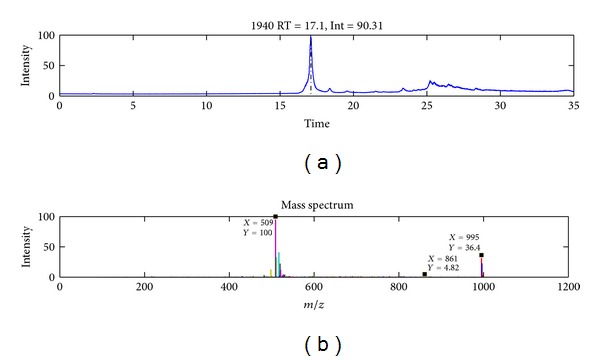
Total ion chromatogram (TIC) of pure MCYST-LR (10 *μ*g/mL) and its spectrum (below) in peak maximal intensity for a retention time of 17.09 min. In the spectrum, the molecular ion at a *m/z* 995 [M + H]^+^, the doubly charged sodium adduct ion at a *m/z* 509 [M + H + Na]^2+^, and the molecular ion with cleavage of the Adda moiety at a *m/z* 861 [M + 2H − 135]^+^ can be observed. The plot was obtained using EMP (Expertomica metabolite profiling) software. The position of the labels is given by the software.

 The chromatographic peak for MCYST-LR was observed at a retention time of approximately 17.1 min under the gradient conditions described previously (Figure 1). The most intense peak observed within the mass spectrum for the given retention time is that of the dicationic sodium adduct ion [M + H + Na]^2+^ (*m/z* 509.2), and the next most intense peak is that of the protonated molecular ion [M + H]^+^ (*m/z* 995.3). The identity of the [M + H + Na]^2+^ was confirmed by manual fragmentation, as the mother ion 509.2 provided MCYST-LR molecular ion 995.3 and dehydrated ion 977.5 in the MS2 spectrum (see Figure 8). Also the distribution of 509.2 isotopologues of was *≈*0.5 confirming the double-charged ion. The cleavage of the Adda moiety (an amino acid unique to all cyanobacterial hepatotoxins) is also visible in the mass spectrum by the presence of an ion with an *m/z* of 861.5 [M + 2H − 135]^+^ (Figure 1). The *m/z* values for the molecular ion and the doubly charged sodium adduct were found to vary for different samples. For the pure MCYST-LR standard, the average *m/z* values were 995.6 and 509.3 for [M + H]^+^ and [M + H + Na]^2+^, respectively. In the analysis of the mixture of MCYST-LR and the extract of *Stigeoclonium*, the observed *m/z* values were 995.7 and 509.2 for [M + H]^+^ and [M + H + Na]^2+^, respectively. Finally, for the mixed samples of MCYST-LR and salmon hydrolyzate, the *m/z* values were 995.1 and 509.2 for [M + H]^+^ and [M + H + Na]^2+^, respectively. The mass shifts in the measured MS spectra of MCYST-LR are under the precision limit of the low-res ion trap (±0.46 *m/z*) used. The low resolution of the mass value is not relevant for the purpose of this paper.

*(i) Responsivity Function*. For the computation of the calibration curves, the [M + H + Na]^2+^ and [M + H]^+^ ions were selected. To determine the responsivity, calibration curves were fitted for both pure and mixed MCYST-LR samples. In the ideal case, within a certain concentration range, the responsivity is assumed to be a linear function as follows: (*y* = *s*∗*c* + *o*) where *c* is the concentration, *y* is the detector response (maximal intensity or area), *s* is the slope of the linear curve, and *o* is the offset. However, in all of our measurements, the dependence of the detector response on concentration was found to have different linear slopes for low and high concentrations. Therefore, as a nonlinear fitting function, the exponential function was selected. The exponential function is given as *y* = *a*∗exp⁡(*b*∗*c*) + *o*, where *a* is the vertical exponential scale, *b* is the horizontal exponential scale, *c* is the concentration, and *o* is the offset. 

**Figure 2:**
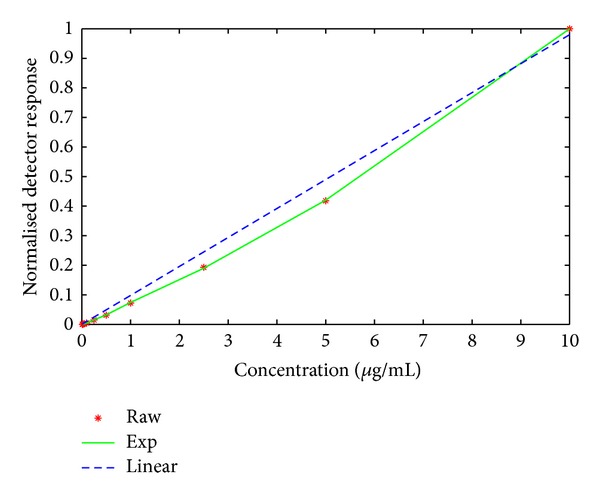
Linear and exponential fittings of concentration-response dependence for the peak area of the pure MCYST-LR molecular ion of *m/z* 995 [M + H]^+^. The red stars indicate the peak areas evaluated by automatic analysis from raw measurements, the blue dashed line indicates the linear function fitting, and the green solid line indicates the exponential fitting function.

 Calibration curves were reconstructed using linear and exponential functions for pure MCYST-LR in MeOH (10 concentrations in duplicates), MCYST-LR in *Stigeoclonium* extract (7 concentrations in triplicates), and MCYST-LR in salmon hydrolyzate (5 concentrations in triplicates). The two lowest concentrations of the analyte (0.01 and 0.025 *μ*g/mL) in salmon hydrolyzate were not detected in any of the three replicates. The measured datasets obtained from the replicates (for each sample type) were averaged to obtain more statistically robust data. The data of interest (averaged maximal intensity and peak area) for each ion were then normalized to the maximal value to avoid the digital arithmetic issues of overflow and underflow. Both functions (linear and exponential) were fitted using Matlab cftool with the evaluation of root mean square error (RMSE) serving as the criterion function. Even though the concentration-response dependencies were close to the linear curve; the exponential curves fit the processed data with a smaller RMSE in almost all of the cases considered (Figure 2). The observed deviation of the calibration data from linearity in ion trap MS has been previously reported [21]. The linear function only provided a better fit for those concentration curves reconstructed from the maximal intensity of the molecular ion and from only three observations (Table 1). Although the differences between the RMSE of the linear and exponential fits are very small (Table 2), this does not imply that the correct fitting function was selected [34]. The existence of a better fitting model cannot be ruled out. 

 In order to examine the responsivity of the three different sample types, we compared the parameters of the fitting functions for all of them. The concentration-response curve parameters exhibited remarkable differences when reconstructed by both linear and exponential functions for pure MCYST-LR and MCYST-LR in *Stigeoclonium *sp. extract and salmon hydrolyzate (Table 2). The linear slopes for the different sample types differ by one order of magnitude (around 0.1 for pure MCYST-LR, 1 for *Stigeoclonium *sp. extract and around 4 for salmon hydrolyzate for normalized data). These differences indicate considerable competition of MCYST-LR ions with coeluting compounds of the matrices in the mixed samples. In other words, the matrix used influenced the responsivity of the analyte. The estimation of matrix influence is present in further subsections.

**Figure 3:**
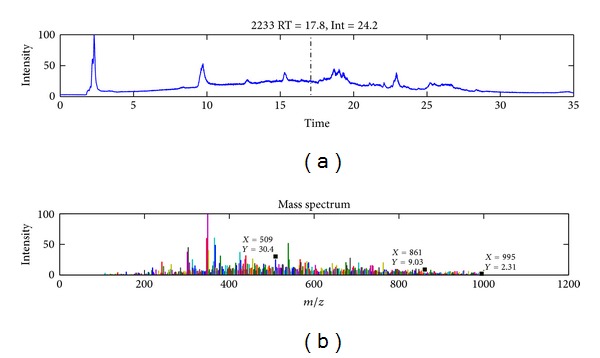
Total ion chromatogram (TIC) of MCYST-LR (0.01 *μ*g/mL) in* Stigeoclonium *sp. extract and its spectrum (below) in peak maximal intensity at a retention time of 17.08 min. In the noisy spectrum, the molecular ion of *m/z* 995 [M + H]^+^, the sodium adduct ion of *m/z* 509 [M + H + Na]^2+^, and the molecular ion with the cleavage of the Adda moiety at an *m/z* 861 [M + 2H − 135]^+^ are still observed. The plot was taken from EMP (Expertomica metabolite profiling) software. The position of the labels is given by the software.

 All measurements were analysed using two methods: (i) manual and (ii) nonparametric analysis. Nonparametric Expertomica metabolomic profiling enabled automatic noise subtraction as well as automatic peak decomposition. Expertomica was able to retain all important ions for microcystin-LR in the* Stigeoclonium *sp. extract at a concentration of 0.01 *μ*g/mL (Figure 3). The probability that the detected ion represents a compound is 95% (Table 3). The other advantage of Expertomica metabolite profiling is that it does not change or recalculate any data values; it only subtracts the non-relevant contributions (noise) according to the estimated probabilities. 

 The PRT reports show relevant information about MCYST-LR in a reasonable way and is simple to be used in postprocessing. Manual data analysis (by Brucker Daltonic DataAnalysis) requires supervised parameterization for data smoothing to reliably integrate the peaks that were manually selected (the *m/z* value and possible shift in mass). Unfortunately, data smoothing changes the intensity values in comparison to the raw data.

*(ii) Limit of Detection*. Because of the mass shift in the given mass spectra precision (±0.46 *m/z*), it was necessary to set the length of mass intervals used for estimation of the statistical parameters. Blank mass axes were downsampled (rounded) to a discriminability level of 1 *m/z* in order to estimate the LOD independently for the target mass values (995 for [M + H]^+^, 509 for [M + H + Na]^2+^, and 861 for [M + 2H − 135]^+^). Three matrix blanks (MeOH, *Stigeoclonium *sp. extract, and salmon hydrolyzate) comprised samples containing no analyte.

 The LOD was computed as the mean blank value of the target mass plus 3 times the blank standard deviation of the target mass. The computed parameters are shown in Table 4. The LOD values increase with the complexity of matrix, as expected. The covariance value is the maximal covariance of the target mass and all other mass values; maximal covariance is the minimal total covariance of the matrix used on the target mass. The correlation and strength values were computed from the maximal covariance values. The matrix offset on the target mass is represented by the value of the correlated responsivity offset as a mean blank value plus correlated standard deviation. 

**Figure 4:**
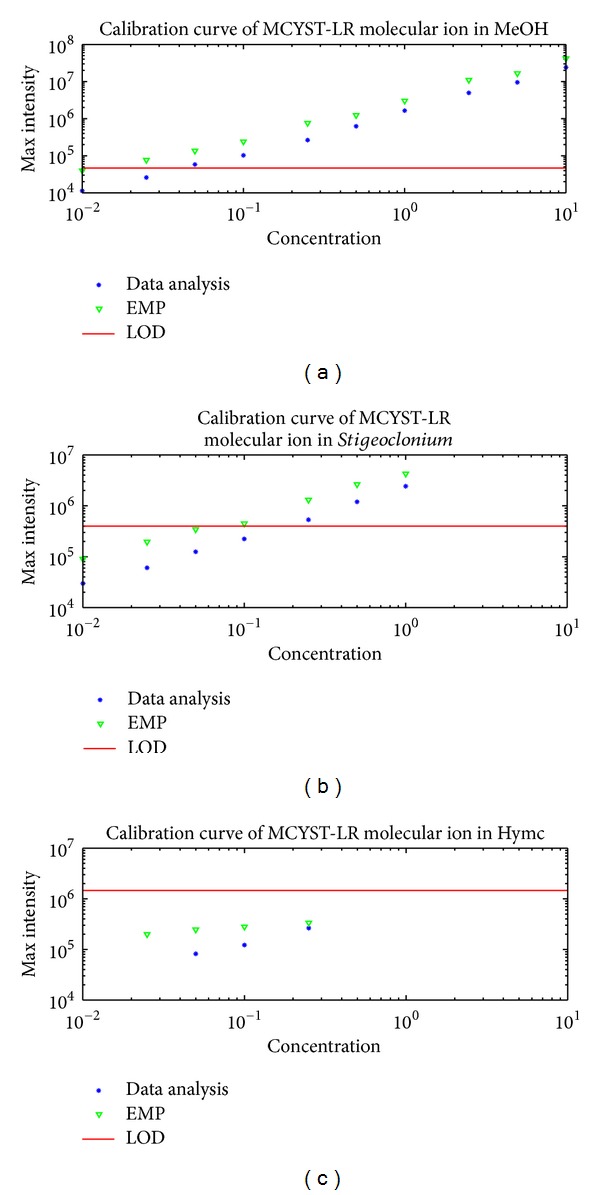
Calibration curves for MCYST-LR in three different matrices (MeOH, *Stigeoclonium *sp. extract, and salmon hydrolyzate) and the computed limit of detection (LOD) for a protonated MCYST-LR molecular ion mass of 995. From (a) to (c) the calibration curve of MCYST-LR in MeOH, the calibration curve of MCYST-LR in *Stigeoclonium *sp.extract, and the calibration curve of MCYST-LR in salmon hydrolyzate. The blue stars are the maximal intensities evaluated by DataAnalysis, the green triangles are the maximal intensities evaluated by EMP (Expertomica metabolite profiling), and the red line is the computed LOD in a given matrix. The calibration curve trend continues below the LOD value. Units for graph axes are follows: *μ*g/mL for the *x*-axis and counts for the *y*-axis.

 Computed LODs were compared with the measured calibration curves for all three matrices (Figure 4). The analyte was detected in concentration levels below the LOD in all three matrices. An interpretation of the situation was proposed by [35]. The true critical level depends on the number and level of interferences in the matrix. The approach to the theoretically predicted critical level (MDL) was derived via multivariate prediction intervals and principal component regression by Boqué et al. [33]. However, there is still no generally accepted multivariate model for the instrument signals detection limit. A similar problem was reported in gamma spectroscopy measurements by Berlizov in 2007 [36]. Berlizov [36] also proposed the “correlation” of LOD according to the background.

 Therefore, univariate LOD represents the basic offset as the blank mean value plus 3 times the univariate (noncorrelated) sensitivity. Therefore, this LOD is the level of false nondetects and should also be interpreted as the univariate offset. The LOD for false detects is better to evaluate as Boqué's multivariate detection limit (MDL). The correlated offset, as a step between the univariate and multivariate approach, is introduced as the correlated responsivity offset, CRO. The CRO value represents the minimal estimation of the matrix contribution to the analyte signal offset via the correlated standard deviation (~correlated sensitivity). Thus, CRO is a useful quantity for ion competition estimation.

*(iii) Estimation of Ion Competition*. The MeOH solution was used as the reference matrix. The calibration curve, including the reference responsivity and transfer function, was measured for 10 different concentrations of MCYST-LR in duplicates. Replicates of blanks of all matrices (MeOH, *Stigeoclonium* sp. extract-Stig, salmon hydrolyzate-Hymc) were also measured. Correlated responsivity offsets CRO1 (MeOH), CRO2 (Stig), and CRO3 (Hymc) as well as correlation coefficients *R*1 (MeOH), *R*2 (Stig), and *R*3 (Hymc) for the target mass values (995 for [M + H]^+^, 509 for [M + H + Na]^2+^, and 861 for [M + 2H − 135]^+^) were estimated for each blank. Data analysis was performed using Expertomica metabolite profiling and Matlab. 

 Reference responsivity (in MeOH) was fitted using Matlab cftool. The RMSE of the responsivity function linear fitting was very small; however, exponential fitting produced a better fit (Table 1) and the difference of the fits was small. Therefore, for the estimation of the short calibration interval (three consecutive concentrations), the linear approximation is sufficient. Linearization on a short interval is approximately equal to the derivative of the exponential responsivity function. The responsivity function for every different triplication has a slightly different linear slope and linear offset. However, the error of linearization on the short interval does not exceed the internal variance of the measurement repetitions for the linearization of the measured calibration curve, although not for the estimation. This is the primary reason that estimation was not done for the whole calibration curve. The estimation was independently computed only on short intervals of three consecutive concentrations of the calibration curve. As the length of the interval increases, the error of linearization increases, and, therefore, the error of estimation will also increase.

 The estimation of ion competition in food additive matrices (Stig and Hymc) was computed via (1) with the knowledge of:

- the reference correlation coefficient, *R*1 of the reference matrix (MeOH),
- the correlation coefficient, *R*2 or *R*3, of the food additive matrix blank (Stig or Hymc),
- the reference correlated responsivity offset, CRO1, of the reference matrix (MeOH),
- the correlated responsivity offset, CRO2 or CRO3, of the food additive matrix blank (Stig or Hymc),
- and the parameters of linear fitting (linear slope *s*1 and linear offset *o*1) of three consecutive concentrations of the MCYST-LR calibration curve in the reference matrix (MeOH).

**Figure 5:**
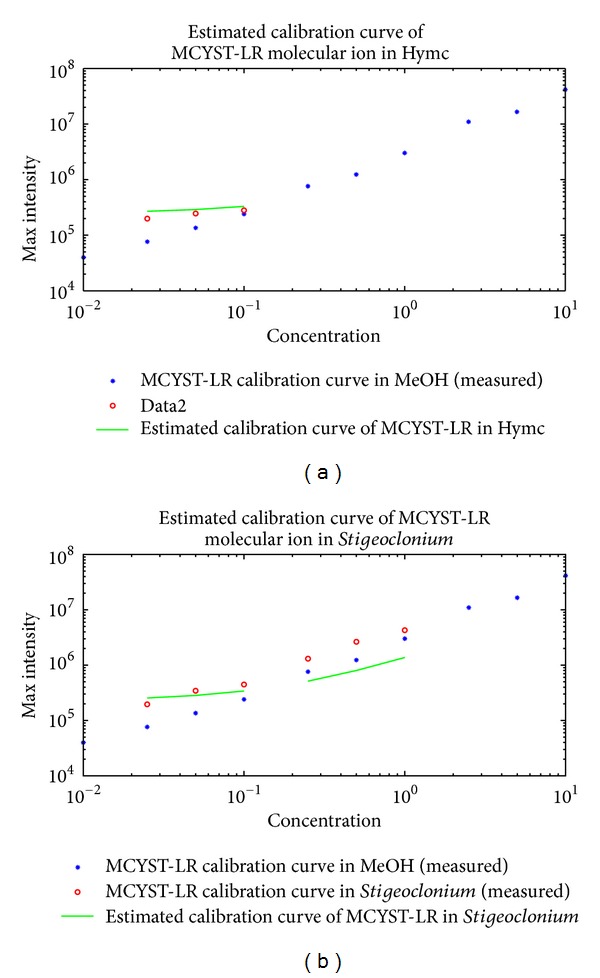
Calibration curves of the protonated MCYST-LR molecular ion in MeOH and estimated calibration curves in food additives matrices (salmon hydrolyzate and *Stigeoclonium *sp. extract). From (a) to (b) estimated calibration curve in salmon hydrolyzate and estimated calibration curve in *Stigeoclonium *sp. extract. The blue stars represent the measured calibration curve of the protonated MCYST-LR molecular ion in MeOH. The red circles represent the measured calibration curve of the protonated MCYST-LR molecular ion in the given food additive matrix. The green lines represents the estimated calibration curve of the protonated MCYST-LR molecular ion in a given food additive matrix. Units for graph axes are follows: *μ*g/mL for the *x*-axis and counts for the *y*-axis.

**Figure 6:**
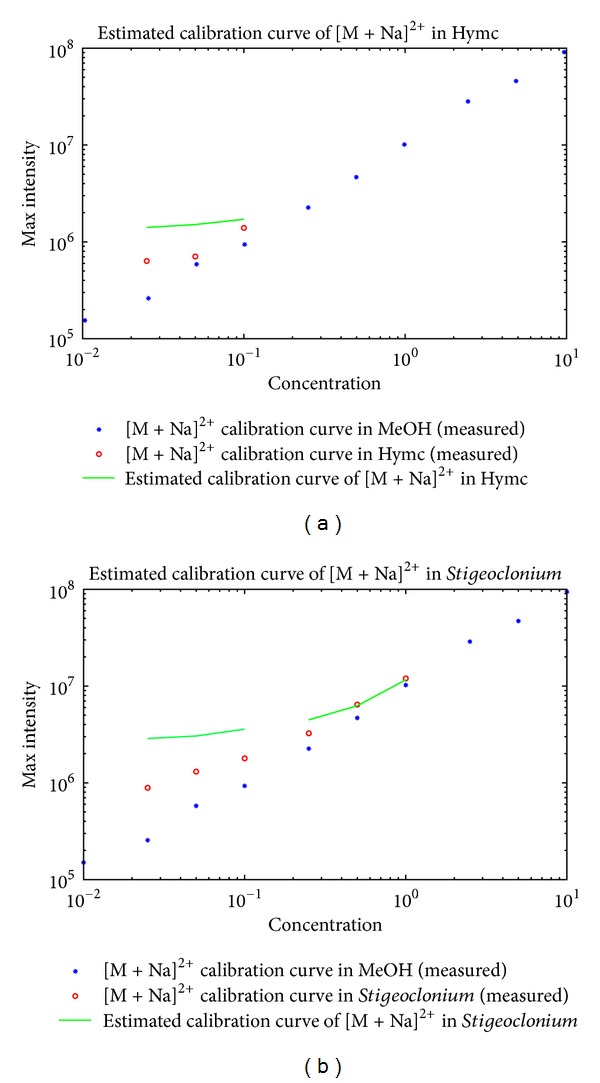
Calibration curves of the doubly charged sodium adduct of the MCYST-LR molecular ion in MeOH and the estimated calibration curves in food additive matrices (salmon hydrolyzate and* Stigeoclonium *sp. extract). From (a) to (b) the estimated calibration curve in salmon hydrolyzate and the estimated calibration curve in *Stigeoclonium *sp. extract. The blue stars represent the measured calibration curve of the doubly charged sodium adduct of the MCYST-LR molecular ion in MeOH. The red circles represent the measured calibration curve of the doubly charged sodium adduct of the MCYST-LR molecular ion in a given food additive matrix. The green lines represent the estimated calibration curve of the doubly charged sodium adduct of the MCYST-LR molecular ion in a given food additive matrix. Units for graph axes are follows: *μ*g/mL for the *x*-axis and counts for the *y*-axis.

**Figure 7:**
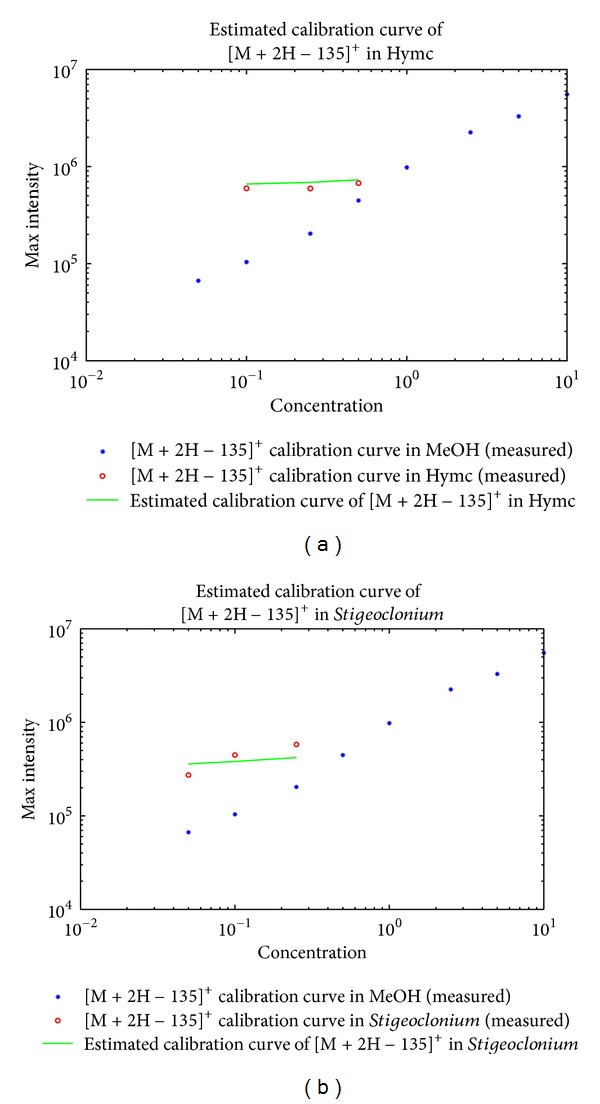
Calibration curves of the MCYST-LR molecular ion with cleavage of the Adda moiety in MeOH and the estimated calibration curves in food additives matrices (hydrolyzate and *Stigeoclonium *sp. extract). From (a) to (b) the estimated calibration curve in salmon hydrolyzate and the estimated calibration curve in *Stigeoclonium *sp. extract. The blue stars represent the measured calibration curve of the MCYST-LR molecular ion with cleavage of the Adda moiety in MeOH. The red circles represent the measured calibration curve of the MCYST-LR molecular ion with cleavage of the Adda moiety in a given food additive matrix. The green lines represent the estimated calibration curve of the MCYST-LR molecular ion with cleavage of the Adda moiety in a given food additive matrix. Units for graph axes are as follows: *μ*g/mL for the *x*-axis and counts for the *y*-axis.

**Figure 8:**
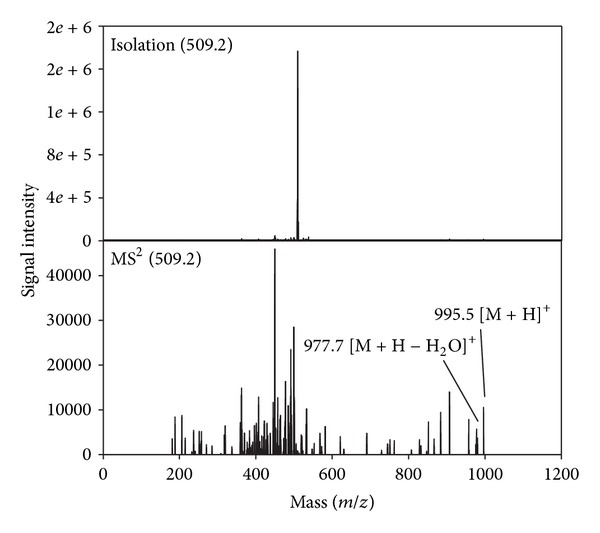
Manual fragmentation, the mother ion 509.2 [M + H + Na]^2+^ provided MCYST-LR molecular ion 995.3 and dehydrated ion 977.5 in the MS2 spectrum.

Results of the estimation are shown in Figures 5, 6, and 7. The interval of the three consecutive concentrations was selected at low concentration values for the three ions, that is, the protonated MCYST-LR molecular ion, doubly charged sodium adduct of the MCYST-LR molecular ion, and the molecular ion with cleavage of the Adda moiety. Three higher consecutive concentrations were also estimated for the* Stigeoclonium *sp. extract for two ions, that is, the protonated MCYST-LR molecular ion and doubly charged sodium adduct of the MCYST-LR molecular ion. 

 The estimated calibration curves for the food additives represent the most probable position of the measured calibration curves according to the matrices offsets and correlations. The exact intensity values are, of course, sample dependent and based on all influences of the matrix on the analyte. Information on the total influence is not present in the blank measurement; therefore, the complete information cannot be estimated before the measurement of the analyte in the matrix is done, at which point the estimation is no longer necessary. However, partial information, that is, correlated responsivity offsets (CROs) and minimal correlations (*R*s), could be computed directly from the blanks. The estimation of the ion competition via CROs and *R*s produces a good approximation of the calibration curve in a given matrix. We are at least able to determine the position where the measured results should be expected. 

 It is quite obvious that some compounds will coelute with the analyte and interfere with ionization. In other words, it is very predictable that matrices may influence and compete with analyte ionization by ESI. However, up until now, an evaluation of the influence of matrices was not possible. In this paper, we propose a method for estimating the competition of analyte hepatotoxin MCYST-LR ions in the measurement of calibration curves in food additives by HPLC-MS. The influence of the matrix comprises two major parts.

1. The chemical noise contributes as the offset to the intensity value of target mass values.
2. The matrix composition affects analyte ionization by correlation of the theoretical offset and even more by the correlation of the responsivity slope in the measurement of the calibration curve.

The main advantage of our approach is the evaluation of the minimal correlation given by the matrix to the target mass values. Therefore, we can estimate both the responsivity offset and the correlation of that offset and the calibration (responsivity) slope. This correlation information is directly evaluated from the blanks of the matrices. In combination with the known measurement of the calibration curve in a known matrix, it can be used for the estimation of the position of the calibration curve in other matrices (food additives) that are known only from their blanks.

 There are several disadvantages that require deeper induction. First of all, our estimated correlation does not represent all of the correlations in the matrices. Correlation is computed as the maximal correlation of the target mass and all of the other masses. From the statistics, it is known that this computed correlation is just the minimal total correlation. The value of the total correlation should be computed via the recurrence equation as the multiplication of primitive and partial correlation coefficients, if those are known. Unfortunately, this is still just the correlation to the matrix noise at the target mass and not to the analyte ion. Correlation of all analyte ions cannot be performed until the measurement is done. However, once the measurement is done, no estimation of the correlation is required.

 The situation of the unknown precision of the total correlation to the analyte ions means that the estimated responsivity slope and real measured responsivity slope will differ. The slope of the responsivity should be slightly higher or lower, which leads to the important point: the estimated calibration curve cannot be extrapolated. The estimated values are valid only in the short interval of linearization. The additional error contribution to extrapolation is the nonlinearity of the responsivity function.

 The correlated responsivity offset, CRO, is computed from the blank noise mean and the correlated sensitivity (correlated standard deviation) of that noise. The CRO is useful for low concentrations above the critical limit of detection (MDL). However, it is expected that the “strength” of the analyte amount will influence the offset during ionization, especially for very high concentrations. The magnitude of the effect of this influence remains unknown.

 Therefore, the exact values of correlation and offset are sample dependent and should be performed only via experiments. On the other hand, the estimation of the minimal influence is hidden in the blank measurements of the matrices. Once again, it is the matrix noise influence and not the total correlation of the analyte. Even so, it is the best approximation of the responsivity and, therefore, of the ion competition and the calibration curve, which should be easily revealed with available knowledge. Therefore, the matrix blank represents the minimal required set of information. Estimation of ion competition via the correlated responsivity offset offers a simple approach for the evaluation of the probable position of the calibration curve in a given matrix. This method is derived directly from the basic properties of the theory of measurement.

## 4. Conclusions

The change in responsivity of pure MCYST-LR and mixtures of MCYST-LR in complex biological samples indicates the influence of coeluting compounds. The phenomenon of ion competition in MS (ESI) has been discussed previously in the literature. In our study, the type of responsivity function (calibration curve) was tested and the exponential function was fitted to the measured calibration curves. For small intervals of three consecutive concentrations, it is sufficient to use approximation via a linear function. 

 We confirmed that the standard limit of detection (LOD) approach typically leads to the neglect of data points that are well within the range of the response curve. With the knowledge of the blanks' mean value and correlated standard deviation (sensitivity), we proposed a method for evaluating the correlated responsivity offset (CRO) for individual target masses of the analyte ions in any given matrices. This value should be used for the estimation of quantitative ion competition among different analytes when they are ionized at the same retention time. 

 The evaluation is valid only for congruent measurement conditions, including the device settings, mobile phase composition, and gradient changes. Agreement between the theoretical and experimental values is sufficient. The proposed algorithm of the correlated responsivity estimation is computationally easy and is promising for wider usage in LC-MS. However, further investigation and verification of additional multivariate responsivity properties remain our focus.

## Figures and Tables

**Table 1:** Evaluation of root mean square errors (RMSEs) for linear and exponential fittings. Fittings were done for concentration-response dependences for areas and maximal intensities of the MCYSTLR molecular ion of *m/z* 995 [M + H]^+^ and sodium adduct ion of *m/z* 509 [M+H+Na]^2+^. Manually and automatically obtained values were fitted for all samples (pure MCYSTLR and MCYSTLR in *Stigeoclonium *sp. extract and salmon hydrolyzate).

		Expertomica			Manual	
	Calibration	Salmon	Stigeoclonium	Calibration	Salmon	Stigeoclonium
Linear RMSE						
Area NA adduct	0.008977	0.128800	0.025770	0.012140	0.058260	0.047040
Area molecular ion	0.028660	0.082940	0.041840	0.027280	0.147200	0.040940
Max intensity NA adduct	0.018420	0.142500	0.126200	0.022360	0.034610	0.021190
Max intensity molecular ion	0.009455	0.038150	0.069690	0.033760	0.015690	0.015250
Exp RMSE						
Area NA adduct	0.008033	0.080920	0.022590	0.007450	0.034090	0.036680
Area molecular ion	0.001540	0.048970	0.020280	0.008426	0.097570	0.037800
Max intensity NA adduct	0.017030	0.091840	0.129800	0.010750	0.023110	0.021510
Max intensity molecular ion	0.009579	0.034330	0.075650	0.010090	0.004033	0.014200

**Table 2:** Slopes of the linear fittings and exponents for the exponential fittings. Fittings were carried out for concentration-response dependences for the areas and maximal intensities of the MCYSTLR molecular ion of *m/z* 995 [M + H]^+^ and the sodium adduct ion of *m/z* 509 [M+H+Na]^2+^. Manually and automatically obtained values were fitted for all samples (pure MCYSTLR and MCYSTLR in *Stigeoclonium *sp. extract and salmon hydrolyzate).

		Expertomica			Manual	
	Calibration	Salmon	Stigeoclonium	Calibration	Salmon	Stigeoclonium
Linear slope						
Area NA adduct	0.10060	4.98100	0.97450	0.10080	3.49100	0.90970
Area molecular ion	0.09800	4.76000	1.03900	0.09802	3.20800	0.98200
Max intensity NA adduct	0.10010	4.37200	0.86500	0.10160	3.99900	0.92120
Max intensity molecular ion	0.10080	3.62500	0.93860	0.09746	3.47800	0.99960
Exp exponent						
Area NA adduct	−0.01077	1.00100	−0.31260	−0.02181	1.00100	−0.69510
Area molecular ion	0.06452	0.99960	−0.67920	0.05913	1.00200	−0.66400
Max intensity NA adduct	−0.02063	1.00400	1.26000	−0.04363	1.00100	0.09998
Max intensity molecular ion	−0.00655	0.99900	0.36720	0.07418	0.99950	0.15920

**Table 3:** Example of lines in the Expertomica metabolite profiling software PRT report for detected peaks of the MCYSTLR molecular ion of *m/z* 995 [M + H]^+^, the sodium adduct ion of *m/z* 509 [M+H+Na]^2+^, and the molecular ion with cleavage of the Adda moiety ion of *m/z* 861 [M + 2H − 135]^+^ for the measurement of MCYSTLR (0.01 *μ*g/mL) in* Stigeoclonium *sp. extract. The MCYSTLR molecular ion was detected with a probability factor of 0.952 (95.2% of the analyte signal). Expertomica metabolite profiling software also reports the relative content of the MCYSTLR molecular ion in the whole measurement, the values of the peak borders (start time and end time) in the time axis as well as the time of maximal intensity (RT), value of that maximal intensity, and peak area.

	Mass	Probability	Relative content	RT	Begin time	End time	Max intensity	Area
Peak	509.2	0.005	0.05847	17.1129	16.9146	17.5930	1693675	2.6462*e* + 007
Peak	861.0	0.952	0.00296	17.0323	17.0179	17.1732	260411	1.3385*e* + 006
Peak	995.7	0.952	0.00062	17.0323	17.0323	17.0395	256928	2.7929*e* + 005

**Table 4:** Computed statistical parameters of target mass values (995 for [M + H]^+^, 509 for [M + H + Na]^2+^, and 861 for [M + H − 135]^+^) in three different blanks (MeOH, *Stigeoclonium* extract: Stig, and salmon hydrolyzate: Hymc). MCYST-LR was not present in the blanks. The parameters are computed only for MS detector ability to measure the noise in the target mass values. Limits of detection (LODs) are independently computed for each mass value using a common equation: mean plus 3 times the standard deviation. Correlated responsivity offsets (CROs) were calculated using the mean and correlated standard deviation. The CRO values differ from the LOD (noncorrelated) values. Correlation may increase or decrease the offset represented by the limits. The strength of relation represents the percentage of the correlation. The strengths have higher values in extract blanks instead of in the MeOH blank, as was expected. The strength of Hymc is the largest according to the more complex, and therefore more correlated, matrix of salmon hydrolyzate.

Blank	MeOH			Stig			Hymc		
Ion	995	509	861	995	509	861	995	509	861
LOD	4.7745**e** + 004	3.9121*e* + 005	9.6128*e* + 004	3.8310**e** + 005	4.0951*e* + 006	6.4347*e* + 005	1.4633**e** + 006	1.9763*e* + 006	1.8746*e* + 006
Covariance	1.6214*e* + 009	6.0132*e* + 010	4.1243*e* + 009	1.3408*e* + 011	5.8271*e* + 012	2.1560*e* + 011	5.9066*e* + 011	9.6831*e* + 011	9.6757*e* + 011
Correlation	0.1953	0.5972	0.2759	0.3723	0.6806	0.4428	0.5574	0.6037	0.5133
Strength of relation	3.8%	35.7%	7.6%	13.9%	46.3%	19.6%	31.1%	36.4%	26.3%
Correlated standard deviation	4.0266*e* + 004	2.4522*e* + 005	6.4221*e* + 004	3.6617*e* + 005	2.4139*e* + 006	4.6432*e* + 005	7.6854*e* + 005	9.8403*e* + 005	9.8365*e* + 005
Mean	8.4699*e* + 003	8.1244*e* + 004	2.2197*e* + 004	5.1823*e* + 004	5.9748*e* + 005	1.1366*e* + 005	2.1932*e* + 005	3.6980*e* + 005	3.0985*e* + 005
CRO	4.8736**e** + 004	3.2646*e* + 005	8.6418*e* + 004	4.1799**e** + 005	3.0114*e* + 006	5.7798*e* + 005	9.8786**e** + 005	1.3538*e* + 006	1.2935*e* + 006
